# The blue brain project: pioneering the frontier of brain simulation

**DOI:** 10.3934/Neuroscience.2023024

**Published:** 2023-11-02

**Authors:** Arosh S. Perera Molligoda Arachchige

**Affiliations:** Faculty of Medicine, Humanitas University, Milan, Italy

Unlocking the mysteries of the human brain stands out as one of the most captivating scientific endeavors of the 21st century. In the last few decades, several inspirational projects have showcased groundbreaking scientific efforts and their potential impact on our understanding of complex systems such as the human brain. One such project is the Blue Brain Project, which is spearheaded by Henry Markram at the École Polytechnique Fédérale de Lausanne (EPFL) and has emerged as a groundbreaking scientific initiative since its inception in 2005. With its ambitious goal of creating a biologically accurate simulation of the human brain, the project has attracted attention from scientists, philosophers, and the general public alike [1].

Containing billions of neurons and trillions of connections, the human brain remains one of the most complex and enigmatic systems known to humankind. Understanding the intricacies of brain function, cognition, and consciousness has long been a fundamental challenge in neuroscience. The Blue Brain Project carries immense significance as it endeavors to unravel these mysteries. By simulating the brain's structure and function, this project holds the potential to revolutionize our understanding of neurological disorders, inform the development of novel therapies, and shed light on the nature of consciousness itself [2].

**Figure 1.**
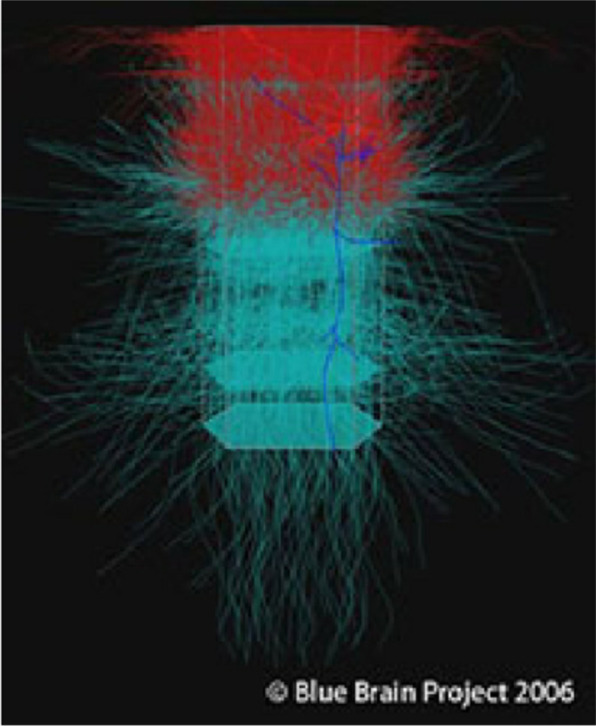
A cortical column simulation with axons and dendrites.

Over the years, the Blue Brain Project has achieved remarkable milestones, thereby advancing our knowledge of brain function. By combining experimental data from neuroscience research with sophisticated computational modeling techniques, the project has succeeded in constructing detailed models of specific brain regions. These models provide a deeper understanding of the electrical and chemical activity of neurons, synaptic plasticity, and neural coding. One notable achievement is the simulation of a rat neocortical column, which is a fundamental unit of the mammalian brain. [3],[4]. By capturing the behavior of thousands of interconnected neurons within the column, the Blue Brain Project demonstrated the power of computational modeling to replicate the emergent properties and dynamics observed in real neural networks. See Figure 1 [5],[12]. This milestone marks a significant step towards the project's ultimate goal of simulating larger brain regions and, potentially, the entire human brain.

Nevertheless, the Blue Brain Project raises critical ethical questions that must be carefully addressed. In the pursuit of groundbreaking scientific initiatives such as the Blue Brain Project, it is imperative to consider not only the remarkable advancements in our understanding of the human brain, but also the environmental impact associated with such endeavors. As a massive undertaking in computational neuroscience, the carbon footprint of the Blue Brain Project is a significant concern. This initiative, which relies on powerful supercomputers and energy-intensive data centers, generates substantial carbon emissions in the course of its operations. While the project holds enormous promise in unraveling the mysteries of the human brain, the associated carbon emissions should not be overlooked. It is crucial to address this environmental impact and explore strategies to mitigate it, thereby aligning the pursuit of scientific knowledge with a commitment to environmental responsibility. This balance between scientific goals and environmental stewardship will be pivotal in ensuring the sustainability and ethical conduct of projects of this magnitude [6].

Another central concern is the potential emergence of consciousness within the simulated brain models. While the project's primary focus is on replicating the brain's structure and function, the question of whether conscious experience could arise within these simulations remains unresolved. This raises ethical dilemmas related to the creation and treatment of potentially conscious entities within the virtual realm. Striking the right balance between scientific exploration and ethical considerations is crucial to ensure responsible and accountable research practices. Another ethical consideration involves the project's reliance on animal experimentation to gather data and validate the models. Animal research has played a significant role in advancing our understanding of the brain; however, it also raises concerns regarding animal welfare and the ethics of using sentient beings for scientific purposes. Rigorous ethical oversight, transparency, and a commitment to minimizing animal suffering are essential aspects that must be upheld throughout the project [7].

Looking ahead, the Blue Brain Project holds tremendous promise for expanding our understanding of the brain. As its computational power continues to advance, the project is poised to simulate larger brain regions and potentially move towards creating whole-brain models. Such advancements could enable unprecedented insights into the brain's functionality, network dynamics, and the mechanisms underlying complex cognitive processes. Furthermore, collaboration with other research initiatives, such as the Human Brain Project, fosters synergy and collaboration among scientists and institutions working towards a common goal of unraveling the mysteries of the brain. By sharing resources, knowledge, and expertise, these collaborations can accelerate progress, facilitate data integration, and provide a more comprehensive understanding of the brain's complexity. Additionally, the Blue Brain Project holds significant implications for the development of artificial intelligence (AI) and brain-inspired computing. The ability to simulate the brain's structure and function could provide valuable insights for designing more efficient and powerful AI systems. By mimicking the brain's neural networks and information processing strategies, researchers can potentially create AI models that better emulate human cognition and exhibit advanced capabilities such as pattern recognition, decision-making, and learning [7].

Despite the significant achievements of the Blue Brain Project, several challenges and limitations must be acknowledged. The complexity of the human brain and the vast amount of data required for accurate simulations present both computational and technological hurdles. Creating a comprehensive and biologically accurate model of the entire human brain remains an immense undertaking that will require further advancements in computational power, data acquisition, and modeling techniques. However, data acquisition will be facilitated by the current advances that neuroimaging is currently undertaking, such as the development of multimodality and ultra-high-field MRI systems, as well as due to the increased integration of AI into radiology [8],[9],[10]. Another challenge lies in the validation and verification of the simulations. While the project strives for accuracy, it is essential to ensure that the simulated models faithfully represent real-life biological systems. Validating the simulated results against experimental data, addressing uncertainties, and continuously refining the models are critical aspects in maintaining scientific rigor and confidence in the project's findings. Moreover, the Blue Brain Project faces funding and resource constraints, as well as the need for collaboration and knowledge-sharing within the scientific community. Securing long-term financial support and fostering partnerships with other research institutions, industry stakeholders, and policymakers will be crucial to sustain the project's momentum and address the complex challenges it entails [11].

The Blue Brain Project stands at the forefront of neuroscience research as a pioneering endeavor, holding immense potential to transform our understanding of the human brain. Its achievements in simulating brain regions and shedding light on fundamental aspects of brain function are commendable. However, ethical considerations surrounding consciousness emergence and animal experimentation must be thoughtfully addressed to ensure responsible scientific progress. As the project moves forward, it will be crucial to tackle the technical, computational, and validation challenges, alongside fostering collaborations and interdisciplinary partnerships. With continued advancements in computational power, increasing data availability, and advancements in neuroscientific research, the Blue Brain Project has the potential to revolutionize our understanding of the brain and pave the way for groundbreaking advancements in medicine, artificial intelligence, and our understanding of the complexities of the human mind.
